# Integrative GWAS and transcriptomics reveal *GhAMT2* as a key regulator of cotton resistance to *Verticillium wilt*

**DOI:** 10.3389/fpls.2025.1563466

**Published:** 2025-04-25

**Authors:** Long Wang, Yonglin Yang, Jianghong Qin, Qifeng Ma, Kaikai Qiao, Shuli Fan, Yanying Qu

**Affiliations:** ^1^ College of Agriculture, Xinjiang Agricultural University, Urumqi, Xinjiang, China; ^2^ Institute of Cotton Research, Chinese Academy of Agricultural Sciences, Anyang, Henan, China; ^3^ Institute of Western Agriculture, Chinese Academy of Agricultural Sciences, Changji, Xinjiang, China; ^4^ Cotton Research Institute, Shihezi Academy of Agricultural Sciences, Shihezi, China

**Keywords:** upland cotton, genome-wide association study (GWAS), *Verticillium dahliae*, ammonium transporter, weighted gene co-expression network analysis (WGCNA)

## Abstract

**Introduction:**

Verticillium wilt, incited by the soilborne fungus *Verticillium dahliae*, is a severe threat to global cotton (*Gossypium* spp.) production, resulting in significant yield losses and reduced fiber quality.

**Methods:**

To uncover the genetic and molecular basis of resistance to this devastating disease, we combined genome-wide association study (GWAS) and transcriptomic analyses in a natural population of 355 upland cotton accessions.

**Results:**

GWAS identified a stable major-effect quantitative trait locus (QTL), *qVW-A01-2*, on chromosome A01, which harbors the candidate gene *GhAMT2*, encoding a high-affinity ammonium transporter. Transcriptomic profiling revealed that *GhAMT2* was significantly upregulated at 12 hours post-inoculation with *V. dahliae*, coinciding with the activation of immune signaling pathways. Weighted Gene Co-expression Network Analysis (WGCNA) further linked *GhAMT2* to critical defense pathways, including lignin biosynthesis, salicylic acid signaling, and reactive oxygen species (ROS) homeostasis, suggesting its role in cell wall reinforcement and systemic immune responses. Functional validation through virus-induced gene silencing (VIGS) confirmed that silencing *GhAMT2* compromised disease resistance. In contrast, transgenic Arabidopsis plants overexpressing *GhAMT2* exhibited enhanced resistance to *V. dahliae*, demonstrating its essential role in defense regulation.

**Discussion:**

These findings establish *GhAMT2* as a key regulator of cotton resistance to Verticillium wilt and highlight its potential for marker-assisted breeding and genetic engineering to improve disease-resistant cotton varieties.

## Introduction

Cotton (*Gossypium* spp.) is a globally important crop, providing approximately 35% of the natural fibers used in the textile industry and serving as a primary renewable source for textile production (Miao et al., 2019; Man et al., 2022). Beyond its primary role in fiber production, cottonseed represents a valuable byproduct, serving as a key resource for edible oil extraction and a high-quality component in animal feed. Cotton is cultivated in over 80 countries worldwide, with around 30 nations relying on it as a significant cash crop in their agricultural economies. According to the United States Department of Agriculture (USDA), global cotton production reached 25.34 million tons during the 2022–2023 season. China leads in raw cotton production, followed by India, the United States, Brazil, and Pakistan, with outputs of 6.1 million tons, 5.99 million tons, 3.06 million tons, 2.83 million tons, and 0.98 million tons, respectively. Among the cultivated cotton species, upland cotton (*Gossypium hirsutum*) accounts for 95% of global production due to its high yield potential and adaptability, making it a major target for cotton breeding programs (Yang et al., 2023).

Despite its global significance, cotton production is severely constrained by abiotic stresses (e.g., drought and salinity) and biotic stresses (e.g., pests and fungal diseases), with the latter accounting for a substantial proportion of production losses. Among these, fungal diseases are particularly devastating, accounting for nearly two-thirds of all infectious plant diseases (Shaban et al., 2018). *Verticillium wilt*, caused by the soilborne fungus *Verticillium dahliae*, is one of the most destructive cotton diseases. Under favorable temperature and humidity conditions, *Verticillium dahliae* produces white spores on infected cotton plants, facilitating the rapid spread of Verticillium wilt and often leading to the swift death of the plant (Fradin and Thomma, 2006). Furthermore, V*. dahliae* can invade cotton bolls and seeds, with infected seeds acting as a major vector for pathogen dissemination, compounding the challenges of disease management. Following infection, *V. dahliae* significantly compromises cotton fiber quality, leading to marked reductions in key parameters such as micronaire value (up to 30% decrease) and fiber length, severely impacting industrial processing and economic returns (Zhang et al., 2012). In China, Verticillium wilt has become a major obstacle to sustainable cotton production, and its control remains a significant challenge.

Resistance to Verticillium wilt in cotton involves a sophisticated network of molecular mechanisms, including structural modifications, hormonal signaling, and the activation of antifungal defense pathways. These mechanisms include structural modifications, the accumulation of antifungal compounds, the activation of hormonal signaling, and the regulation of ROS homeostasis (Song et al., 2020; Wu et al., 2022a). Key genes implicated in these processes have been identified through extensive research. For example, genes such as *GhPGIP1*, *GhPMEI3*, and *GhRFS6* enhance cell wall integrity by modulating pectin metabolism, which inhibits *V. dahliae* colonization (Liu et al., 2017a, Liu et al., 2017b; Chang et al., 2023). The deposition of lignin, callose, and suberin in vascular tissues, regulated by genes like *GhLAC15* and *GhWRKY1-like*, acts as a physical barrier to pathogen invasion (Guo et al., 2016; Zhang et al., 2019; Chen et al., 2021). Additionally, the accumulation of antifungal metabolites, including flavonoids and gossypol, regulated by genes such as *GhSNAT1* and *GhWRKY41*, further suppresses fungal growth (Li et al., 2019; Xiao et al., 2023). Plant immunity is also reinforced through pattern-triggered immunity (PTI) and effector-triggered immunity (ETI), mediated by receptor-like proteins such as Ve1 and nucleotide-binding site leucine-rich repeat (NBS-LRR) proteins like GbRVd (Fradin et al., 2011; He et al., 2018; Li et al., 2021b). Hormonal pathways, especially those involving jasmonic acid (JA) and salicylic acid (SA), are pivotal in mediating cotton’s immune responses. For example, genes such as *GhPLP2* and *GhERF6* regulate JA biosynthesis and ethylene signaling, contributing to enhanced resistance (Yang et al., 2015; Zhu et al., 2021). Furthermore, ROS homeostasis is maintained by genes such as *GhRbohD* and *GbNRX1*, which balance ROS production and scavenging to prevent oxidative damage while promoting defense responses (Chang et al., 2020; Huang et al., 2021).

Numerous studies have utilized GWAS to identify candidate genes associated with critical agronomic traits in cotton (Su et al., 2016; Li et al., 2021a; Wang et al., 2023; Li et al., 2024); however, research focusing on *Verticillium wilt* resistance remains relatively limited. The advent of genomic tools, including GWAS and transcriptome analysis, has revolutionized our ability to unravel the genetic basis of Verticillium wilt resistance, enabling the identification of key resistance loci and candidate genes. GWAS, in particular, leverages the genetic diversity of natural populations to identify loci associated with resistance, eliminating the need for traditional biparental crossing designs (Zhang et al., 2020). For instance, Abdelraheem et al. applied GWAS to a natural population of 376 upland cotton accessions, successfully confirming previously known QTLs related to resistance and uncovering novel candidate regions (Abdelraheem et al., 2022). Recent investigations have identified key quantitative trait loci (QTLs) and candidate genes involved in resistance mechanisms, including transcriptional regulation, cell wall reinforcement, secondary metabolism, and hormone signaling pathways (Zhao et al., 2021). Nevertheless, despite these advancements, a comprehensive understanding of the molecular mechanisms underpinning resistance remains elusive, emphasizing the need for further research in this area.

In this study, we employed a combined approach of GWAS and transcriptomic analysis to investigate the genetic architecture of Verticillium wilt resistance in a natural population of 355 upland cotton accessions, which were sourced from major cotton-growing regions both within China and internationally. High-quality SNPs obtained from whole-genome resequencing were used to identify QTLs and candidate genes associated with resistance. Transcriptome analysis further elucidated the functional roles of these genes in response to *V. dahliae* infection. This study provides a comprehensive understanding of the genetic and molecular mechanisms of Verticillium wilt resistance in cotton and offers valuable genetic resources for developing resistant cotton varieties. The findings are expected to accelerate the breeding of cotton cultivars with enhanced resistance to *Verticillium wilt*, contributing to sustainable cotton production in the face of this devastating disease.

## Materials and methods

### GWAS population and field experiments

The study utilized 355 upland cotton (*Gossypium hirsutum* L.) germplasm resources, representing diverse ecological regions as previously reported (Li et al., 2021a). The germplasm accessions were systematically evaluated for their resistance to Verticillium wilt under controlled field conditions, with experiments designed to minimize environmental variability and ensure consistent disease pressure. The cotton natural population was planted at Anyang (36 08’N, 114 48’E) and phenotypically evaluated for *Verticillium wilt* resistance across three consecutive years (2016–2018) under field conditions, designated as E4 (2016), E5 (2017), and E6 (2018), to capture variations in environmental conditions and disease severity. Each site represented a unique environment, providing a robust basis for evaluating resistance across varying conditions. The experiments were performed using a randomized complete block design with three replications to ensure reliability and minimize environmental variability.

### Phenotyping and statistical analysis of disease index

The phenotypic data from three environments were analyzed using R software (v 4.3.2). Descriptive statistical analyses, including mean, standard deviation, skewness, and kurtosis, were performed using the “pastecs” package, and the normality of the data was assessed with the Shapiro-Wilk test. Best linear unbiased prediction (BLUP) values for each phenotype were estimated under three environments and across combined growing conditions using the “lme4” package (Bates, 2014). Frequency distribution histograms were created using the “ggplot2” package to visualize the phenotypic data distribution across environments (Wickham, 2011). The disease index (DI) was calculated using the formula as following: $\text{DI}=\frac{\sum \left(\text{ni}\times \text{ri}\right)}{\text{N}\times \text{R}}\times 100$ , where *n_i_* is the number of plants at the *i-th* disease severity level, *r_i_​* is the corresponding disease severity score, *N* is the total number of plants, and *R* is the maximum disease severity score. The calculated DI values were incorporated into subsequent statistical analyses, including BLUP estimation, to evaluate genotype performance under varying environmental and disease conditions.

### Genome-wide association study of *Verticillium wilt* resistance trait

GWAS was conducted using the mixed linear model (MLM) implemented in GEMMA (v0.98.3) (Zhou and Stephens, 2012). The analysis included a total of 2,262,367 high-quality single nucleotide polymorphisms (SNPs). Phenotypic data comprised Verticillium wilt disease index values measured across three environments, along with BLUP values calculated using multiple approaches. Batch processing for GWAS was streamlined with the use of vcf2gwas software (v0.8.7) (Vogt et al., 2022). To refine the results, SNPs were filtered based on linkage disequilibrium (LD) block intervals using the GEC software (Li et al., 2012). After filtering, 45,681.10 SNPs were retained for downstream analysis. The significance threshold for association was determined using the Bonferroni correction, calculated as -log_10_(*P*) > 5.96.

### Genetic diversity analysis and candidate gene identification

To estimate nucleotide diversity (π), VCFtools (v0.1.16) was utilized (Danecek et al., 2011). Manhattan and QQ plots were generated using the R package “CMplot” (https://github.com/YinLiLin/CMplot). Linkage disequilibrium (LD) block analysis was performed based on the LD decay principle of upland cotton using the “LDheatmap” package (Shin et al., 2006). Haplotype analysis was conducted employing the “ggplot2” package in R.

### RNA-seq experimental workflow and analysis

To elucidate the molecular basis of cotton’s resistance to Verticillium dahliae, RNA-seq experiments were conducted using two cotton varieties: Zhongzhimian 2 (ZZM2), a resistant cultivar, and Jimian 11 (JM11), a susceptible cultivar. Root samples were collected at 13 distinct time points post-inoculation to capture the dynamic gene expression changes associated with the plant’s immune response. These time points included 0 hours (prior to inoculation), 1 hour, 3 hours, 6 hours, 9 hours, 12 hours, 24 hours, 48 hours, 72 hours, 96 hours, 120 hours, 144 hours, and 168 hours post-inoculation. Total RNA was extracted from these samples using the FastPure Plant Total RNA Isolation Kit (Vazyme Biotech, Nanjing), designed to handle samples with high polysaccharide and polyphenol content. The integrity and quality of the RNA were assessed through spectrophotometry and agarose gel electrophoresis to confirm suitability for downstream applications. High-quality RNA was used to construct sequencing libraries, following a standardized workflow, with sequencing performed on an Illumina platform. The resulting data underwent stringent quality control processes, including the removal of low-quality reads and adapter sequences. Metrics such as Q30 values and GC content were analyzed to ensure high reliability.

### Co-expression network analysis and enrichment

Gene co-expression networks were constructed using Weighted Gene Co-expression Network Analysis (WGCNA) implemented in R software (v4.2.1). Gene expression patterns from resistant and susceptible varieties at 13 time points were analyzed via PCA to determine major components driving the expression variance. Differentially expressed genes were further analyzed for functional enrichment using GO and KEGG analyses via the Biomarker Cloud Platform (https://www.biocloud.net/). These tools enabled the identification of key biological pathways and molecular functions associated with resistance to *V. dahliae*.

### Virus-induced gene silencing and pathogen inoculation

To investigate the functional role of *GhAMT2* in cotton resistance to *Verticillium dahliae*, a virus-induced gene silencing (VIGS) system was employed. The VIGS vector *pYL156-GhAMT2*, containing a specific fragment of the *GhAMT2* gene from Xinshi K28 variety, was constructed and co-transformed with *pYL192* into cotyledons of 10-day-old cotton seedlings via Agrobacterium-mediated infiltration. An empty vector *pYL156* (TRV: EV) served as a negative control, while *pYL156-GhCLA1*, inducing a bleaching phenotype, acted as a positive control to validate the VIGS system. One-week post-infiltration, plants displaying the bleaching phenotype (positive control) were sampled, and qRT-PCR confirmed the silencing efficiency of *GhAMT2* using the 2^−ΔΔCt^ method. For pathogen inoculation, the *Verticillium dahliae* strain Vd991 was cultured on potato dextrose agar (PDA) and subsequently expanded in Czapek-Dox broth. After filtering to remove hyphae, spores were adjusted to 1 × 10^7 spores/mL. Cotton seedlings with fully expanded second true leaves were inoculated by applying 10 mL of the spore suspension to the soil surface. Plants were maintained under controlled greenhouse conditions (25°C, 65% relative humidity) for disease development. Disease symptoms were assessed 25 days post-inoculation, and the disease index was calculated to evaluate the phenotypic differences between silenced and control plants, thereby elucidating the role of *GhAMT2* in cotton resistance to *V. dahliae*.

## Results

### Field phenotypic evaluation of *Verticillium wilt* resistance in natural population of upland cotton

Descriptive statistical analysis of Verticillium wilt resistance traits across the three environments (E1–E3) revealed considerable variation in the disease index (DI), with DI values ranging from 30.90 to 33.21, indicating significant phenotypic diversity among the tested accessions. Overall, the 355 upland cotton germplasm lines exhibited substantial variability in DI, with coefficients of variation ranging from 44.61% to 50.39% (Additional File 1: **Supplementary Table S1**). The frequency distribution histograms of DI across the three field environments showed an approximately normal distribution pattern (Additional File 2: **Supplementary Figure S2**). Further variance analysis indicated that both genotype and environment significantly influenced the DI of the 355 cotton lines (Additional File 1: **Supplementary Table S2**). This suggests that genetic factors predominantly contribute to DI variation, making these materials well-suited for GWAS.

### GWAS analysis of verticillium wilt resistance in upland cotton

In this study, a total of 2,262,367 high-quality SNPs were obtained by aligning and filtering resequencing data from 355 upland cotton accessions. Using a linear mixed model (LMM), a GWAS was conducted on the *Verticillium wilt* DI observed under field conditions and the BLUP values (**Supplementary Figure S1**). A significance threshold of -log10(P) = 5.96 was set, identifying 376 significant loci grouped into 6 QTL intervals (**Table 1** and Additional File 1: **Supplementary Table S3**). Among these, one QTL, *qVW-A01-2*, was consistently detected across multiple environments.

**Table 1:** Significant QTLs associated with *verticillium wilt*.

QTLs	Position	SNP	-log_10_(*P*)	PVE (%)	Environments
*qVW-A01-1*	A01	9731094	6.58	7.21	E1
*qVW-A01-2*	A01	117626096	10.35	11.52	E1/E/E3/BLUP
*qVW-A05-1*	A05	2903716	6.28	6.85	BLUP
*qVW-A07-1*	A07	27984793	6.13	6.69	BLUP
*qVW-A13-1*	A13	108087914	6.97	7.66	E2/BLUP
*qVW-D12-1*	D12	61677556	8.32	9.22	E2/E3/BLUP

### Identification and haplotype analysis of the *Ghir_A01G022270* associated with *Verticillium wilt* resistance in upland cotton

**Figure 1:**
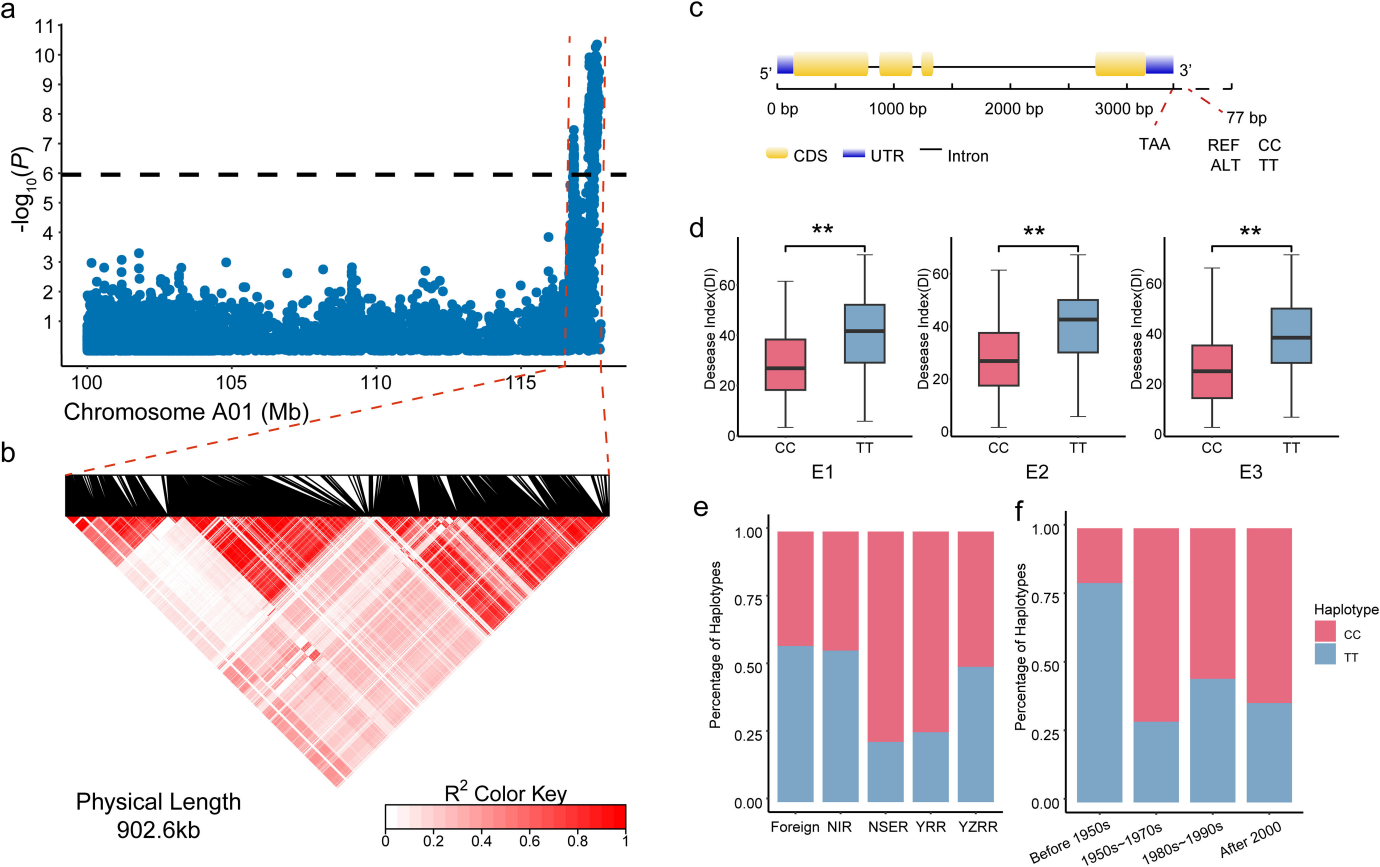
Identification of candidate genes within the QTL interval qVW-A01-2. **(a)** Local Manhattan plot of chromosome A01, with the QTL interval qVW-A01-2 highlighted between the red dashed lines; **(b)** Linkage disequilibrium (LD) heatmap of the 902.6 kb candidate region within the QTL interval; **(c)** Gene structure of the candidate gene *Ghir_A01G022270*; **(d)** Boxplot illustrating the disease index phenotypes of the two haplotypes of *Ghir_A01G022270* across environments E1 to E3 (**P < 0.01); **(e)** Statistical frequency distribution of *Ghir_A01G022270* haplotypes across different ecological regions; **(f)** Statistical frequency distribution of *Ghir_A01G022270* haplotypes across different breeding periods.

A stable and significant QTL, *qVW-A01-2*, was identified on chromosome A01 (**Figure 1a**). Linkage disequilibrium (LD) analysis determined the QTL interval to span 902.6 kb (**Figure 1b**), containing 95 genes. Among these, a candidate gene, *Ghir_A01G022270*, was identified. This gene belongs to the AMT gene family, encoding a high-affinity ammonium transporter and is expressed in buds and roots. Located on the negative strand of chromosome A01, a significant SNP, rsGhir_A01_117556376, was identified 77 bp downstream of the gene (**Figure 1c**). This SNP was found to be significant in both the E3 environment and the BLUP, with -log10(*P*) values of 8.39 and 7.61, respectively. Two haplotypes, CC and TT, were associated with this gene. Among the 355 materials analyzed, 208 carried the CC haplotype, which included accessions from the Yangtze River Valley (24), Yellow River Valley (115), Northwestern Inland (44), Northern Early Maturity (14), and foreign germplasm (11). The remaining 139 materials carried the TT haplotype, distributed among the Yangtze River Valley (40), Yellow River Valley (24), Northwestern Inland (56), Northern Early Maturity (4), and foreign germplasm (15). Haplotype analysis revealed significant differences in disease indices between the two haplotypes across three environments. Accessions carrying the CC haplotype exhibited significantly lower disease indices compared to those with the TT haplotype, indicating that CC is the favorable haplotype for Verticillium wilt resistance (**Figure 1d**). In the Northern Early Maturity and Yellow River Valley cotton regions, the CC haplotype was more prevalent, with frequencies exceeding 75%, unlike the Yangtze River Valley, Northwestern Inland, and foreign germplasm, where the two haplotypes were more evenly distributed (**Figure 1e**). This suggests that resistant varieties dominate in the NSER and YRR regions. Cotton breeding progress was categorized into four periods based on breeding goals and strategies: pre-1950, 1950s–1970s, 1980s–1990s, and post-2000. Analysis of the distribution of the two haplotypes over these periods revealed that the CC haplotype was rare before 1950. Its frequency began to increase significantly from the 1970s onward, coinciding with the emergence and outbreak of Verticillium wilt (**Figure 1f**).

### Transcriptome sequencing and differentially expressed gene identification

**Figure 2:**
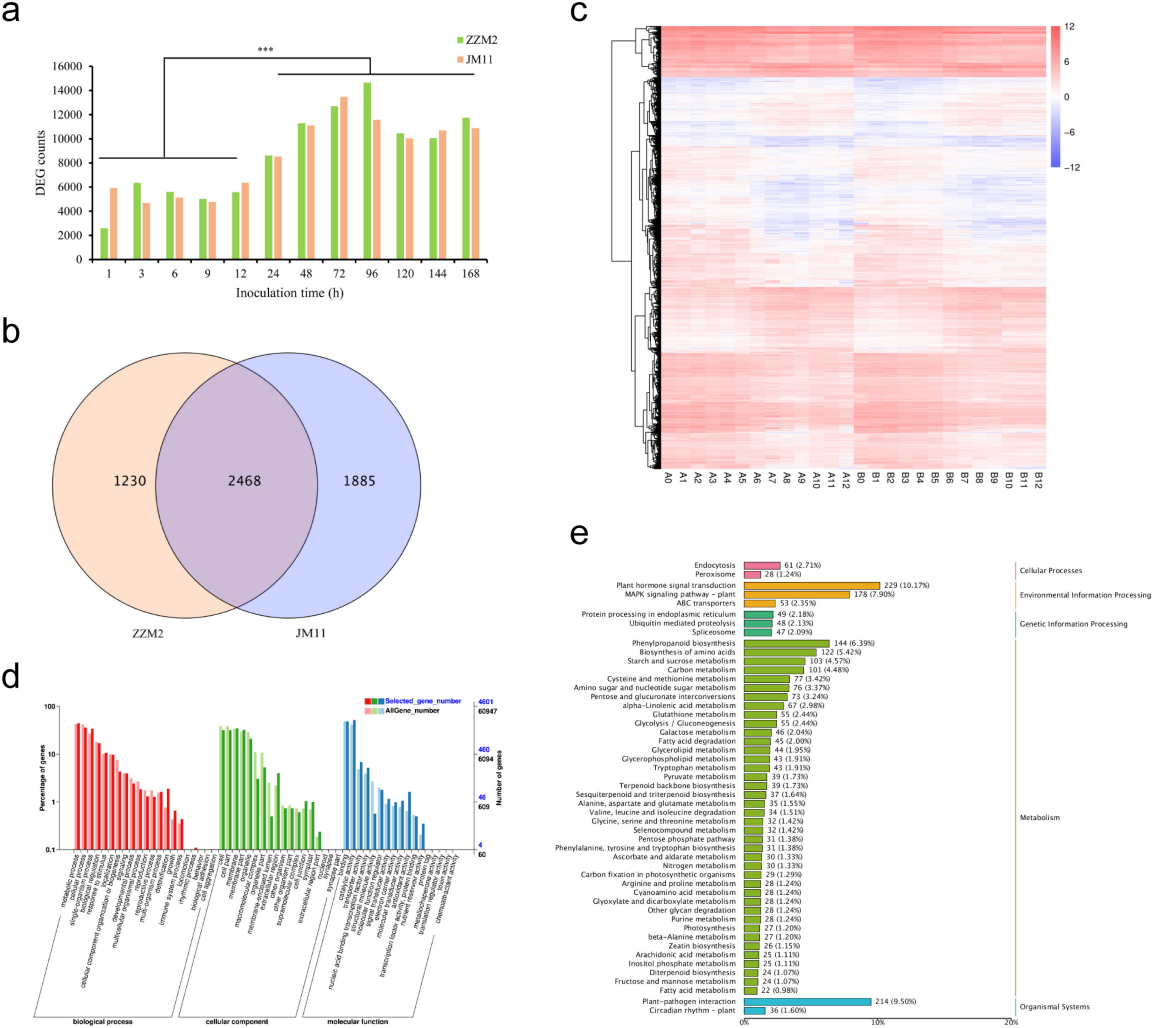
Analysis of differentially expressed genes (DEGs) in response to Verticillium wilt infection. **(a)** Statistics of differentially expressed gene counts at various time points post-inoculation with *Verticillium dahlia*; **(b)** Venn diagram illustrating the overlap of 5,583 differentially expressed genes between Zhongzhi Mian 2 (ZZM2) and Ji Mian 11 (JM11); **(c)** Expression clustering analysis of 5,583 DEGs across 26 samples. A and B represent ZZM2 and JM11, respectively; time points 0 to 12 correspond to 0, 1, 3, 6, 9, 12, 24, 48, 72, 96, 120, 144, and 168 hours post-inoculation; **(d)** Gene Ontology (GO) enrichment analysis of the 5,583 DEGs, highlighting key biological processes, cellular components, and molecular functions; **(e)** KEGG pathway enrichment analysis of the 5,583 DEGs, showing significant pathways associated with plant-pathogen interactions, hormone signaling, and metabolic processes. ***, P < 0.001.

Transcriptome sequencing was conducted on the roots of resistant cotton line Zhongzhimian 2 (ZZM2) and susceptible line Jimian 11 (JM11) at 13 time points post-inoculation with *Verticillium dahliae*: 0 h, 1 h, 3 h, 6 h, 9 h, 12 h, 24 h, 48 h, 72 h, 96 h, 120 h, 144 h, and 168 h. A total of 26 sequencing samples generated Clean Reads ranging from 20,366,019 to 27,118,026, with an average of 22,856,093 reads per sample. The GC content ranged from 43.21% to 43.79%, averaging 43.49%, while the Q30 scores ranged from 93.43% to 95.28%, with an average of 94.90% (Additional File 1: **Supplementary Table S4**). Quality control analysis of the sequencing data produced a total of 177.71 Gb of Clean Data, with an average of 6.83 Gb per sample. Mapping to the *Gossypium hirsutum* TM-1 reference genome revealed a unique mapping rate of 87.01% to 92.01%, with an average of 90.12% (Additional File 1: **Supplementary Table S5**). These results indicate that the transcriptome data are abundant and of high reliability. Transcriptome sequencing revealed dynamic changes in differentially expressed genes (DEGs) over time in both ZZM2 and JM11. From 1 h to 12 h post-inoculation (hpi), the number of DEGs remained relatively low (<200 in both lines). However, DEGs increased sharply after 12 h, peaking at 96 h with 1,230 DEGs in ZZM2 and 1,885 DEGs in JM11, before stabilizing by 168 h (**Figure 2a**). Principal component analysis (PCA) further indicated significant differences in DEG expression between samples collected before 12 h (Group1) and after 12 h (Group2), emphasizing a marked shift in gene expression profiles post-12 h, corresponding to the activation of immune responses to *Verticillium dahliae*. These findings align with previous studies, which suggest that cotton initiates a robust gene expression response to combat Verticillium wilt after 12 h. To identify key genes regulating cotton resistance to *Verticillium wilt*, DEG analysis was performed between Group1 and Group2 for ZZM2 and JM11. In ZZM2, 3,698 DEGs were identified between A_Group1 and A_Group2, including 1,045 upregulated and 2,653 downregulated genes. For JM11, 4,353 DEGs were identified between B_Group1 and B_Group2, with 1,411 upregulated and 2,942 downregulated genes (**Figure 2b**). A Venn diagram analysis of DEGs between ZZM2 and JM11 revealed 2,468 shared DEGs (44.20%). Additionally, ZZM2 had 1,230 unique DEGs (22.03%), while JM11 had 1,885 unique DEGs (33.76%). Clustering analysis of 5,583 non-redundant DEGs across the 26 samples identified two distinct expression patterns. Cluster 1 included genes significantly downregulated in both ZZM2 and JM11 before 12 hpi compared to samples from 24 to 168 hpi. In contrast, Cluster 2 comprised genes significantly upregulated in ZZM2 and JM11 during the same period (**Figure 2c**). These results suggest that these 5,583 DEGs play crucial roles in the cotton response to *Verticillium wilt*. To further understand the molecular functions of these DEGs in cotton resistance, Gene Ontology (GO) enrichment analysis was conducted. The DEGs were enriched in 50 GO categories. In the “Biological Process” category, 2,049 DEGs were associated with “metabolic process,” 1,651 with “cellular process,” and 1,577 with “single-organism process.” Notably, 20 DEGs were annotated under “immune system process.” In the “Cellular Component” category, 1,610 and 1,490 DEGs were related to “membrane” and “membrane part,” while 1,455 DEGs were associated with “cell” and “cell part.” In the “Molecular Function” category, 2,379 DEGs were related to “catalytic activity,” 2,234 to “binding,” and 317 to “transporter activity” (**Figure 2d** and Additional File 1: **Supplementary Table S6**). KEGG pathway analysis showed that the 5,583 DEGs were enriched in 150 metabolic pathways. Key pathways included ‘plant hormone signal transduction’ (229 DEGs), ‘plant-pathogen interaction’ (214 DEGs), and ‘MAPK signaling in plants’ (178 DEGs), all of which are crucial for coordinating immune responses to *Verticillium dahliae* (**Figure 2e**, Additional File 1: **Supplementary Table S7**). These pathways are consistent with those previously reported to be involved in plant immune responses to *V. dahliae*, further supporting the reliability of DEG analysis between Group1 and Group2 for identifying genes associated with *Verticillium wilt* resistance.

#### WGCNA co-expression network analysis and identification of candidate genes for *Verticillium wilt* resistance

**Figure 3:**
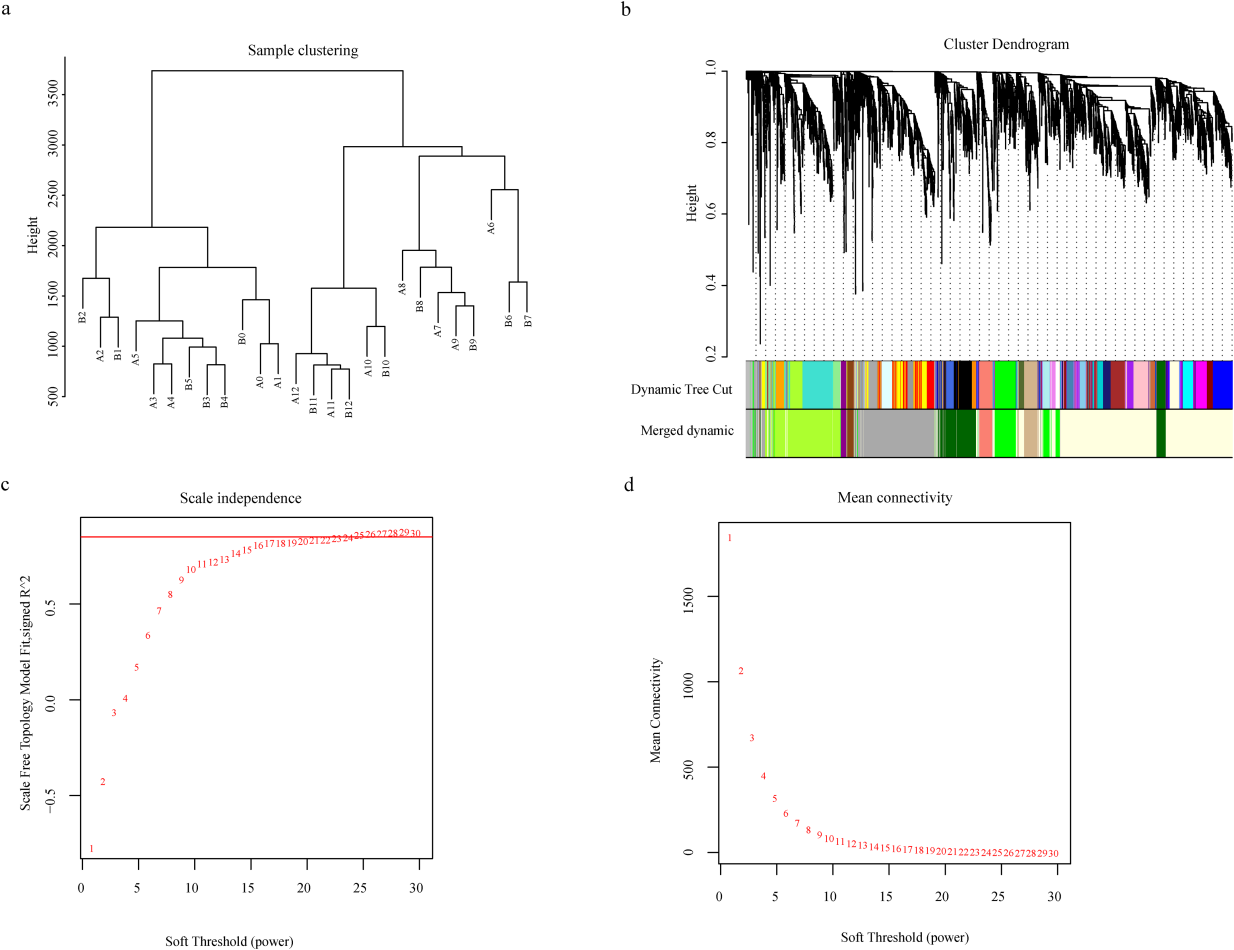
Weighted Gene Coexpression Network Analysis (WGCNA) of 5,583 Differentially Expressed Genes. **(a)** Cluster dendrogram illustrating the global relationships among the 26 samples, highlighting sample clustering patterns; **(b)** Hierarchical dendrogram of gene coexpression modules identified through WGCNA. A total of nine distinct modules were identified, each represented by a unique color based on eigengene calculations; **(c)** Soft threshold analysis showing scale independence to determine the optimal power for network construction; **(d)** Soft threshold analysis of mean connectivity, demonstrating the preservation of network connectivity under varying threshold powers.

Using the 5,583 differentially expressed genes (DEGs) from the 26 samples, Weighted Gene Co-expression Network Analysis (WGCNA) was performed. Sample clustering analysis revealed no outlier samples among the 26 (**Figure 3a**). Analysis of scale independence and mean connectivity determined that the soft threshold was 26, where the scale-free topology fit index (*R²*) first exceeded 0.9 (**Figures 3c, d**). Based on this threshold, the expression patterns of the 5,583 DEGs across the samples were grouped into nine co-expression modules (**Figure 3b**), with the number of genes in each module ranging from 41 (Darkmagenta module) to 1,239 (Lightyellow module). Heatmap analysis of gene expression networks within modules showed that the genes in the Lightyellow module were significantly correlated with genes in multiple other modules. Furthermore, intra-module gene correlation analysis revealed that the Lightyellow, Greenyellow, and Green modules exhibited high gene expression correlation, while the Tan and Saddlebrown modules showed weaker correlations (Additional File 2: **Supplementary Figure S3**). The association between the nine gene modules and *Verticillium wilt* resistance was analyzed (Additional File 2: **Supplementary Figure S4**), and all modules were significantly correlated with resistance (p < 0.05). Among them, the MEgreen, MElightyellow, and MEgrey modules exhibited the highest correlations with Verticillium wilt resistance phenotypes, whereas the MEtan and MEsaddlebrown modules showed lower correlations (Additional File 2: **Supplementary Figure S5**). These results suggest that the module-trait association approach alone may not be sufficient to identify key genes regulating *Verticillium wilt* resistance. Integrating transcriptome and GWAS identified the *GhAMT* (*Ghir_A01G022270*) located within the major-effect QTL (*qVW-A01-2*). This gene showed highly significant differential expression between the early (0–12 hours post-inoculation) and late (24–168 hours post-inoculation) infection stages in both Zhongzhi Mian 2 and Jimian 11. Functional annotation revealed that *Ghir_A01G022270* encodes a high-affinity ammonium transporter closely related to nitrogen metabolism, an essential nutrient for plant growth and development. Efficient expression of ammonium transporters enhances nitrogen utilization, thereby boosting plant vigor and stress tolerance. Previous studies have demonstrated that adequate nitrogen supply can improve disease resistance in plants, suggesting a potential link between the superior performance of *Ghir_A01G022270* and cotton’s resistance to Verticillium wilt. Therefore, we propose *Ghir_A01G022270* as a strong candidate gene for regulating resistance to Verticillium wilt in cotton.

### Functional characterization of *GhAMT2* in regulating *Verticillium wilt* resistance

**Figure 4:**
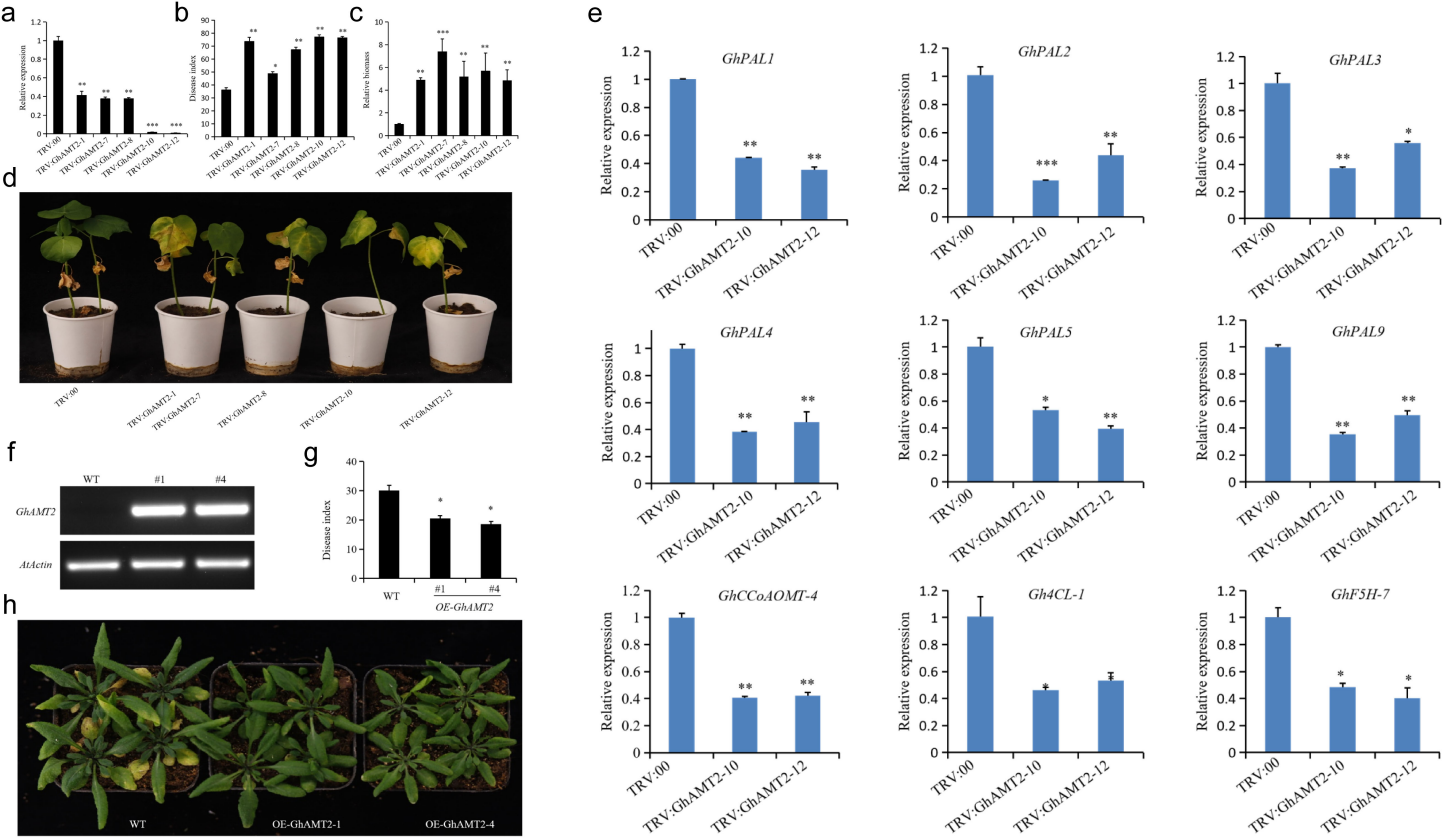
Functional Analysis of *GhAMT2* in enhancing resistance to *Verticillium Wilt* in Cotton and Arabidopsis. **(a)** Relative transcript levels of *GhAMT2* in TRV-VIGS cotton leaves for *TRV: EV* (empty vector) and *TRV: GhAMT2* plants, with *GhActin* serving as the internal reference gene. Expression levels were normalized to “1” for TRV: EV; **(b)** Silencing of *GhAMT2* reduces cotton resistance to *Verticillium dahliae*, as evidenced by increased disease symptoms on *TRV: GhAMT2* plants compared to *TRV: EV* plants 25 days post-infection; **(c)** Statistical analysis of the disease index in *GhAMT2*-silenced plants, showing a significant increase compared to controls; **(d)** Relative biomass of *V. dahliae* in *TRV: GhAMT2* and TRV: EV plants, illustrating higher pathogen colonization in *GhAMT2*-silenced plants; **(e)** *GhAMT2* promotes the expression of lignin metabolism-related genes in cotton. Expression analysis of lignin biosynthesis genes showed upregulation in the presence of *GhAMT2*; **(f)** Identification of transgenic Arabidopsis plants overexpressing *GhAMT2*. Semi-quantitative RT-PCR analysis demonstrates *GhAMT2* mRNA levels in three transgenic lines compared to WT (Col-0); **(g)** Overexpression of *GhAMT2* enhances Arabidopsis tolerance to *Verticillium dahliae*, as evidenced by reduced disease symptoms in transgenic lines compared to WT; **(h)** Statistical analysis of the disease index in WT and OE-*GhAMT2* Arabidopsis plants, showing significantly lower disease indices in transgenic lines. *, 0.01<P<0.05; **, P < 0.01; ***,P < 0.001.

To investigate the role of *GhAMT2* in cotton resistance to *Verticillium dahliae*, we employed virus-induced gene silencing (VIGS) technology for functional validation. A *pYL156-GhAMT2* vector containing a *GhAMT2*-specific fragment was constructed to silence the endogenous *GhAMT2* gene in cotton. The *pYL156-GhAMT2* plasmid was mixed with *pYL192* in a 1:1 ratio and injected into the cotyledons of 10-day-old cotton seedlings via Agrobacterium-mediated transformation. An empty *pYL156* vector served as a negative control (*TRV: EV*), while *pYL156-GhCLA1*, which induces a bleaching phenotype, was used as a positive control to verify the VIGS efficiency. This process generated *GhAMT2*-silenced cotton plants (*TRV: GhAMT2*) alongside the control line (*TRV: EV*). One week post-inoculation, the positive control displayed the expected bleaching phenotype. qRT-PCR analysis confirmed a significant reduction in *GhAMT2* expression in *TRV: GhAMT2* plants compared to *TRV: EV* plants, with transcript levels decreasing by approximately 80% (p < 0.01). These results validate the efficiency of the virus-induced gene silencing (VIGS) system used in this study (**Figure 4a**), demonstrating the effective silencing of *GhAMT2*. Subsequently, both *TRV: GhAMT2* and *TRV: EV* plants were inoculated with *V. dahliae* and cultured. Disease severity was evaluated 25 days post-inoculation by calculating the disease index. Results showed that *TRV: GhAMT2* plants exhibited significantly more severe disease symptoms (**Figure 4b**), a higher disease index, and greater fungal colonization within the plants (**Figures 4c, d**) compared to *TRV: EV* plants. These findings indicate that *GhAMT2* plays a role in cotton resistance to *V. dahliae*, and silencing *GhAMT2* compromises the plant’s resistance to this pathogen.

To elucidate the potential mechanism by which *GhAMT2* regulates cotton resistance to Verticillium wilt, we first analyzed the expression of genes involved in the lignin biosynthesis pathway. Using the *GhAMT2*-silenced plants generated in the VIGS experiments and the control plants, we examined the expression levels of key lignin biosynthesis-related genes. The results revealed that, compared to the control plants, the *GhAMT2*-silenced plants exhibited significantly reduced expression levels of several lignin biosynthesis genes, including *GhPAL1*, *GhPAL2*, *GhPAL3*, *GhPAL4*, *GhPAL5*, *GhPAL9*, *GhCCoAOMT-4*, *Gh4CL-1*, and *GhF5H-7* (**Figure 4e**). These findings suggest that the resistance to *Verticillium dahliae* mediated by *GhAMT2* may involve the activation of the lignin biosynthesis pathway.

To further validate the role of *GhAMT2* in plant responses to *Verticillium dahliae*, we generated transgenic Arabidopsis lines overexpressing *GhAMT2*. The coding sequence of *GhAMT2* was cloned into the entry vector *pQBV3*, and subsequently introduced into the plant expression vector *pEG101* under the control of the 35S promoter via LR recombination, resulting in the construction of the 35S:*GhAMT2* expression vector. This vector was transformed into wild-type Arabidopsis Col-0 (WT) using the Agrobacterium-mediated floral dip method to achieve stable heterologous expression of *GhAMT2*. T0 generation seeds were harvested and screened for positive transformants using 10% Basta herbicide. Homozygous transgenic lines were obtained in the T3 generation through segregation analysis. Two transgenic lines with high *GhAMT2* expression levels (OE-*GhAMT2*-1 and OE-*GhAMT2*-4) were selected for Verticillium wilt resistance assays (**Figures 4f, g**). Two-week-old wild-type (WT, Col-0) and transgenic Arabidopsis plants were inoculated with *V. dahliae*, and phenotypic observations were made two weeks post-inoculation. Disease indices were calculated to assess resistance. The results demonstrated that *GhAMT2* transgenic lines exhibited significantly enhanced resistance to Verticillium wilt, with a markedly lower disease index compared to the WT plants (**Figure 4h**). These findings confirm that *GhAMT2* plays a critical role in enhancing resistance to *V. dahliae*.

## Discussion

### Insights from GWAS for identifying resistance loci to *Verticillium wilt*

Genome-wide association studies (GWAS) have proven indispensable for dissecting the genetic architecture of complex traits like Verticillium wilt resistance. In this study, GWAS pinpointed qVW-A01-2 as a major-effect QTL consistently associated with Verticillium wilt resistance across multiple environments. This locus represents a robust genomic region likely harboring key determinant of resistance. The consistent association of *qVW-A01-2* with Verticillium wilt resistance across diverse environments demonstrates its stability and potential for marker-assisted selection (MAS). Interestingly, the *qVW-A01-2* locus overlaps with genomic regions associated with lignin biosynthesis, hormone signaling, and pathogen recognition, which are critical pathways in plant immunity (Yao et al., 2020; Jones et al., 2024). Such co-localization suggests that this locus may integrate multiple resistance mechanisms to provide broad-spectrum and durable resistance. Previous studies have reported similar findings where stable QTLs linked to hormone-regulated defense pathways enhance resistance to soil-borne pathogens, highlighting the importance of cross-pathway integration in effective defense strategies. Moreover, the GWAS approach allowed us to leverage the natural diversity of the cotton population, capturing rare alleles and uncovering loci that are often missed in biparental mapping studies. This diversity-driven strategy not only broadens the genetic base for breeding but also accelerates the identification of beneficial alleles that can be introgressed into elite cultivars. Future studies should focus on fine mapping and haplotype analysis of qVW-A01-2 to identify the causative variants and explore their functional implications in resistance mechanisms.

### Transcriptomic analysis reveals key candidate genes for *Verticillium wilt* resistance

The integration of transcriptomic data with GWAS has significantly enhanced our ability to identify candidate genes underlying complex traits. In this study, transcriptome sequencing revealed dynamic gene expression changes between resistant (ZZM2) and susceptible (JM11) cotton lines during *Verticillium dahliae* infection. Notably, *GhAMT2* emerged as a prominent candidate within the qVW-A01-2 region, with significant differential expression observed at key infection stages, particularly after 12 hours post-inoculation, when the immune response becomes pronounced. Weighted Gene Co-expression Network Analysis (WGCNA) further linked *GhAMT2* to co-expression modules enriched for genes involved in lignin biosynthesis, salicylic acid signaling, and reactive oxygen species (ROS) scavenging. These pathways are well-documented for their roles in fortifying cell walls, signaling systemic acquired resistance (SAR), and detoxifying oxidative stress during pathogen attack (Qi et al., 2017; Jeon et al., 2023; Tian et al., 2024). For instance, lignin deposition acts as a physical barrier to inhibit pathogen invasion, while hormonal signaling pathways mediate immune responses (Mine et al., 2018; Lee et al., 2019). The co-expression of *GhAMT2* with genes such as *GhPAL1*, *Gh4CL1*, and *GhCCoAOMT4* highlights its potential role in regulating these critical defense processes. Importantly, the transcriptomic analysis also identified time-dependent changes in the expression of *GhAMT2*, suggesting that its regulatory effects are tightly linked to the progression of pathogen infection. This temporal aspect aligns with the stepwise activation of plant immune responses, from pathogen recognition to the deployment of structural and biochemical defenses. Further functional studies of co-expressed genes within these modules will provide deeper insights into the molecular networks underpinning cotton resistance to *V. dahliae*.

### Functional validation of GhAMT2 confirms its role in *Verticillium wilt* resistance

The functional significance of *GhAMT2* in cotton resistance to Verticillium wilt was unequivocally demonstrated through virus-induced gene silencing (VIGS) and transgenic experiments. Silencing *GhAMT2* resulted in heightened disease susceptibility, characterized by increased disease indices, reduced lignin biosynthesis gene expression, and higher fungal colonization in the vascular tissues. Conversely, transgenic Arabidopsis plants overexpressing *GhAMT2* exhibited a significant enhancement in disease resistance, with a lower disease index compared to wild-type controls. These results confirm the pivotal role of *GhAMT2* in mediating cotton’s defense response. The functional annotation of *GhAMT2* as a high-affinity ammonium transporter highlights its involvement in nitrogen metabolism, a critical process influencing plant growth and defense (Wu et al., 2022b). Nitrogen plays a dual role in enhancing plant immunity: it supports the synthesis of key defense compounds such as lignin and secondary metabolites, and it boosts overall plant vigor, enabling more robust responses to pathogen attack (Thalineau et al., 2016). Previous studies have shown that ammonium transporters enhance nitrogen use efficiency, which is directly linked to improved resistance to various biotic stresses (Luo et al., 2023). The significant differential expression of *GhAMT2* during early (0–12 h) and late (24–168 h) infection stages suggest its dual role in both basal and induced immune responses. Early activation may prime the plant’s defense system, while sustained expression during later stages likely supports prolonged resistance through structural reinforcement and metabolic adjustments. Additionally, the localization of *GhAMT2* within the qVW-A01-2 locus strengthens its candidacy as a key gene contributing to cotton resistance to *V. dahliae*. Future work should focus on elucidating the downstream targets of *GhAMT2* and its interaction with other resistance-related genes to comprehensively understand its regulatory network. These findings provide a solid foundation for incorporating *GhAMT2* into molecular breeding programs aimed at enhancing Verticillium wilt resistance in cotton.

## Data Availability

The datasets presented in this study can be found in online repositories. The names of the repository/repositories and accession number(s) can be found below: https://ngdc.cncb.ac.cn/ , PRJCA035130 https://www.ncbi.nlm.nih.gov/ , PRJNA389777.
