# Supplementary material for: Integrative GWAS and transcriptomics reveal GhAMT2 as a key regulator of cotton resistance to Verticillium wilt
Source: Front Plant Sci. 2025 Apr 25;16:1563466. doi: 10.3389/fpls.2025.1563466 (PMC12062179; doi:10.3389/fpls.2025.1563466)
Supplement: Supplementary file 2 [file Table2.docx]

**
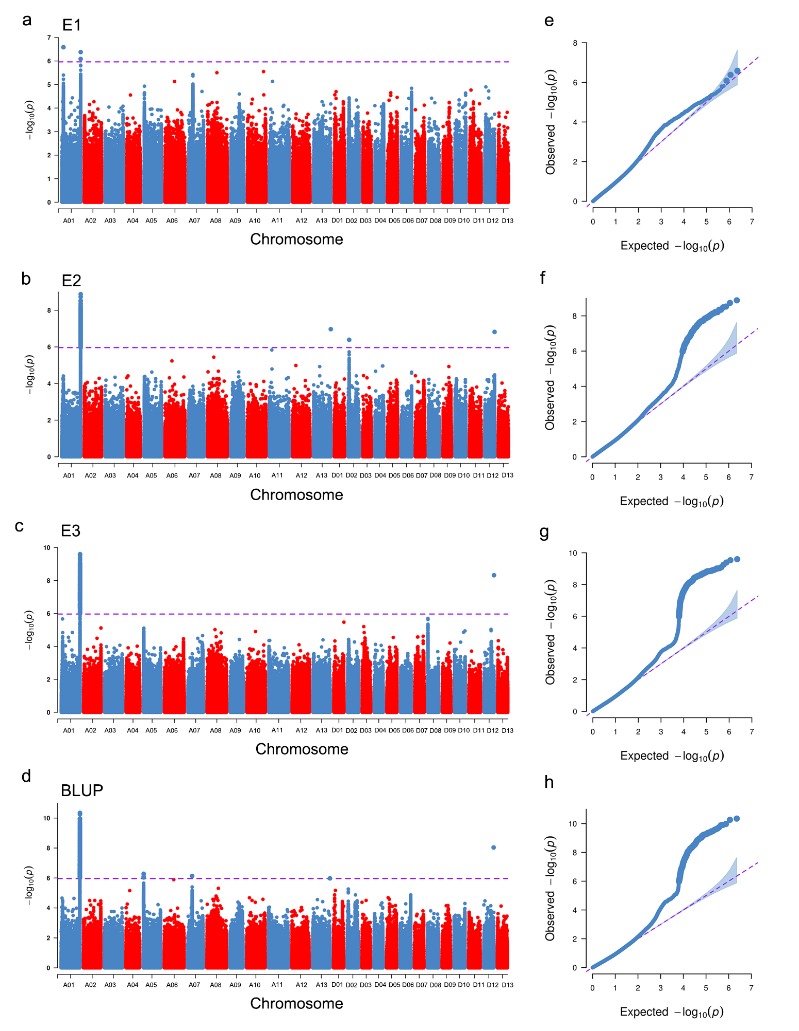
**

**Fig. S1: Manhattan and QQ plots of the disease index and BLUP values for Verticillium wilt across three field environments (2016–2018).** Panels (a-d) illustrate the Manhattan plots for the *Verticillium wilt* disease index over three years (2016, 2017, and 2018), including their respective BLUP values and the overall BLUP values under all planting conditions. The dashed line indicates the genome-wide significance threshold. Panels (e-h) present the corresponding QQ plots for the disease index and its BLUP values, as well as the overall BLUP values for Verticillium wilt across the same three-year period.


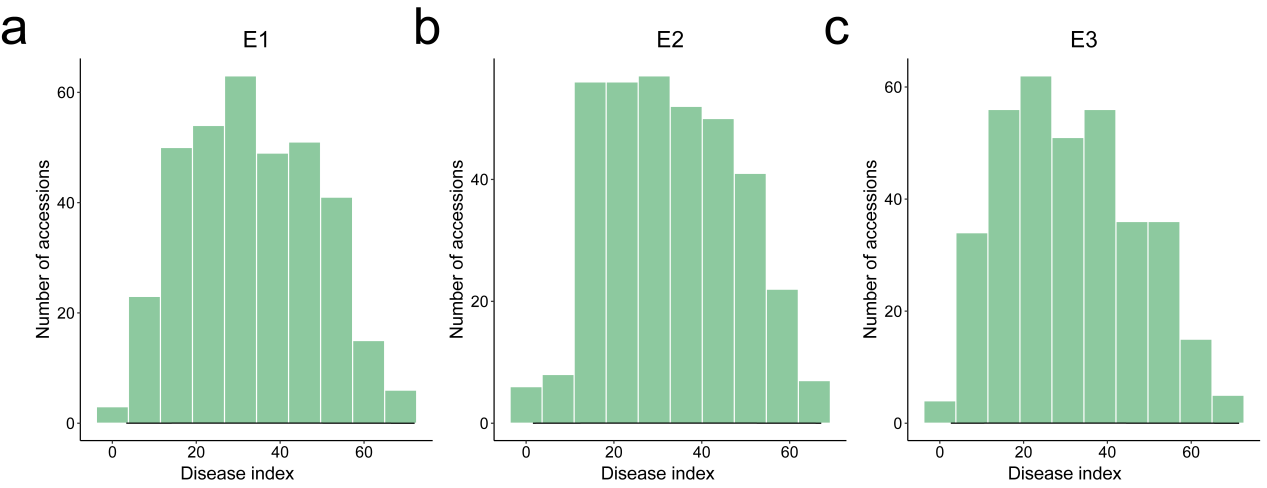


Fig. S2: Phenotypic variation analysis of *Verticillium Wilt* in field (E1: Field-2016; E2: Field -2017; E3: Field -2018).


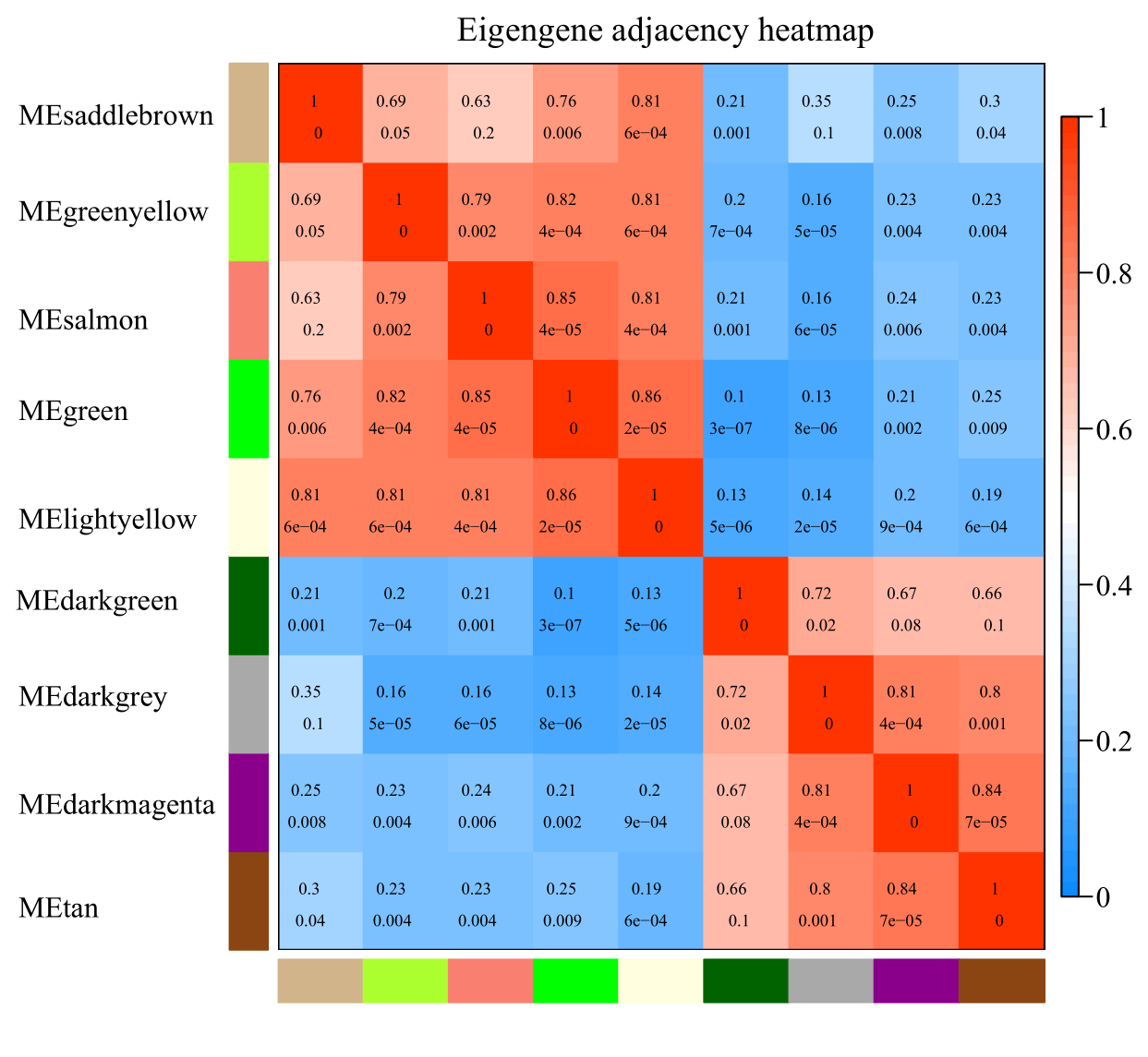


Fig. S3: Network heatmap plot between different modules.


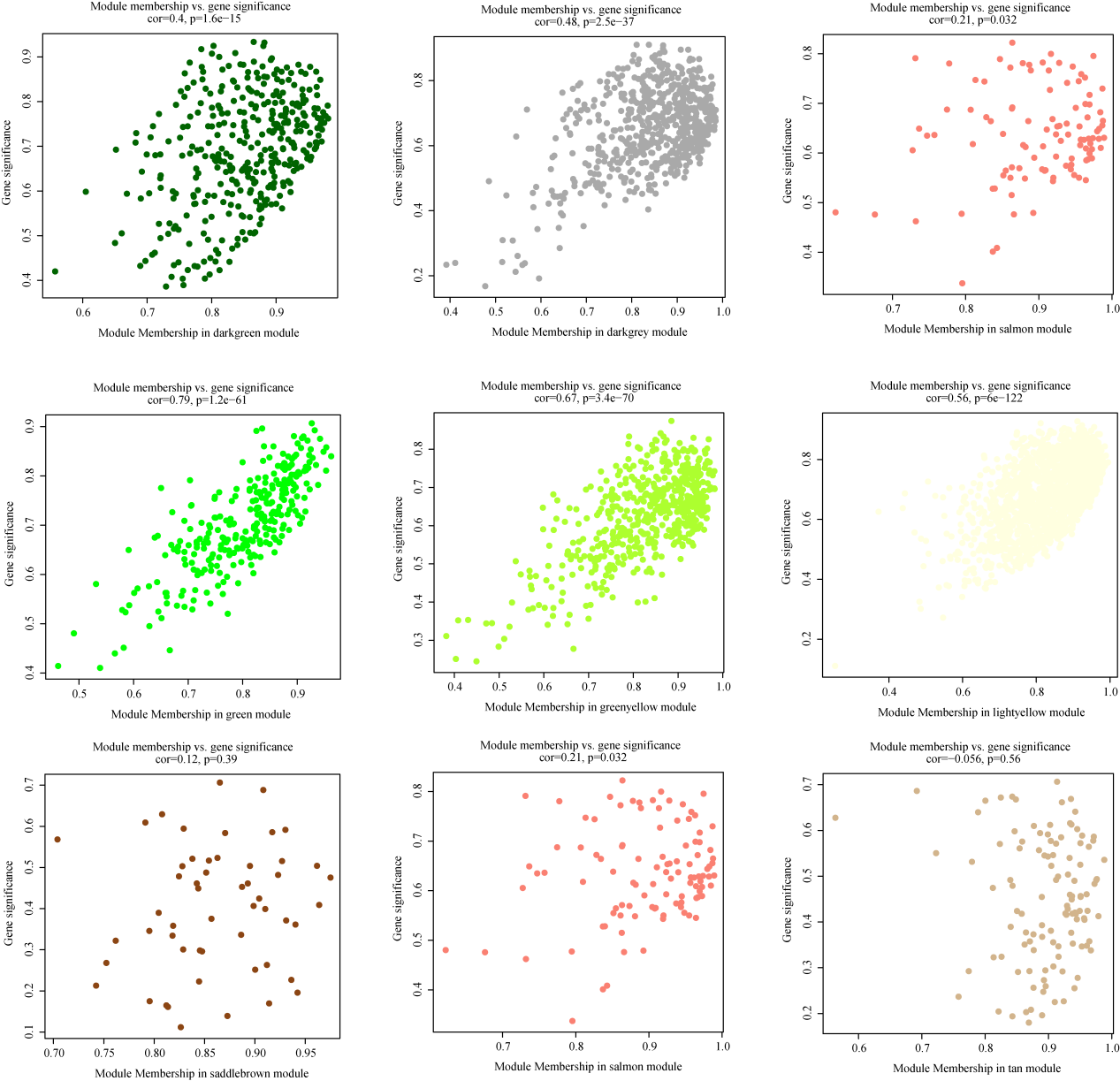


Fig. S4 Expression correlation analysis of genes within 9 modules.


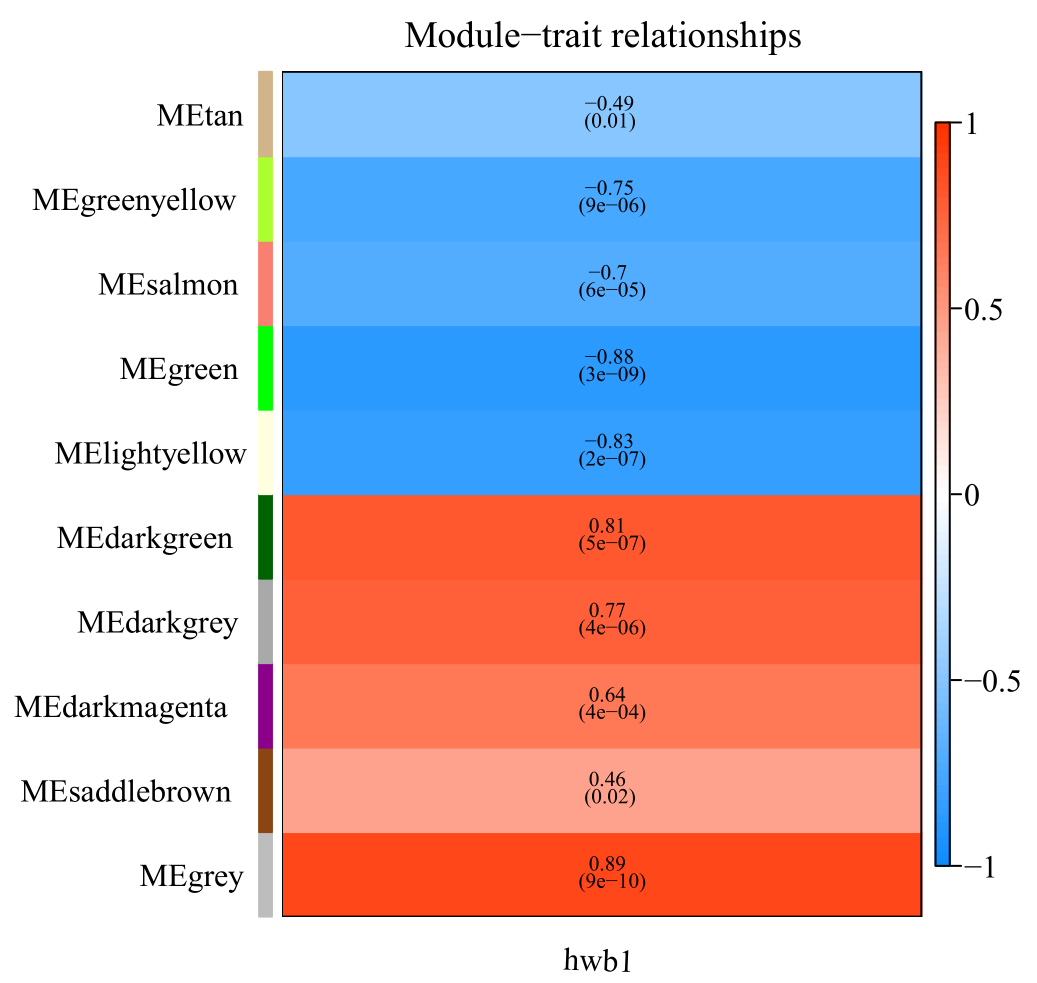


Fig. S5 Correlation analysis of 9 Modules.
